# TPDH-Graphene as a New Anodic Material for Lithium Ion Battery: DFT-Based Investigations

**DOI:** 10.1021/acsomega.4c06252

**Published:** 2024-09-03

**Authors:** Juan Gomez Quispe, Bruno Ipaves, Douglas Soares Galvao, Pedro Alves da Silva Autreto

**Affiliations:** † Center for Natural and Human Sciences, Federal University of ABC, Santo Andre, Sao Paulo 09210-580, Brazil; ‡ Applied Physics Department and Center for Computational Engineering & Sciences, State University of Campinas, Campinas, Sao Paulo 13083-970, Brazil

## Abstract

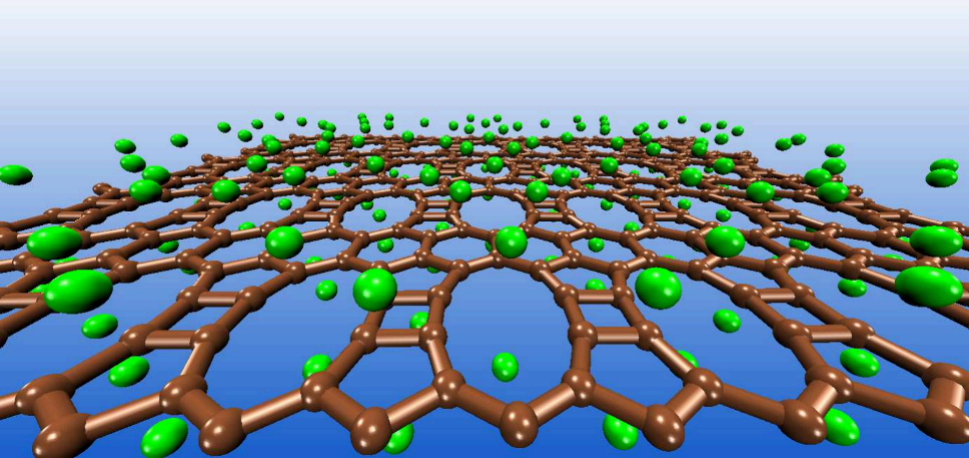

The potential of tetra-penta-deca-hexagonal graphene (TPDH-gr), a recently proposed 2D carbon allotrope as an anodic material in lithium ion batteries (LIBs), was investigated through density functional theory calculations. The results indicate that Li-atom adsorption is moderate (around 0.70 eV), allowing for easy desorption. Moreover, energy barriers (0.08–0.20 eV), diffusion coefficient (>6 × 10^–6^ cm^2^/s), and open circuit voltage (0.29 V) calculations show rapid Li atom diffusion on the TPDH-gr surface, stable intercalation of lithium atoms, and good performance during the charge and discharge cycles of the LIB. These findings, combined with the intrinsic metallic nature of TPDH-gr, indicate that this new 2D carbon allotrope is a promising candidate for use as an anodic LIB material.

## Introduction

Graphene, a revolutionary material, is a two-dimensional (2D) structure composed of a single layer of carbon atoms arranged in a hexagonal honeycomb lattice, where carbon atoms are covalently bonded in *sp*^2^ hybridization.^1^ This hybridization results in excellent mechanical rigidity and high electronic mobility due to the sigma and π type bonds.^2^

In contrast to graphene, other 2D carbon allotropes can be formed by combining different hybridization states (*sp*, *sp*^2^, or *sp*^3^) and different types of carbon rings. One example is graphenylene, which is composed of tetragonal (*C*_4_), hexagonal (*C*_6_), and dodecagonal (*C*_12_) carbon rings.^3^ Calculations based on density functional theory (DFT) indicate that graphenylene has a semiconducting nature with a direct band gap value of approximately 25 meV.^4^ Another carbon allotrope, recently synthesized,^5^ is TPH-graphene, which is formed by *C*_4_, *C*_5_, and *C*_7_ carbon rings, and can exist in two different semiconducting phases with direct band gap values of 2.704 and 2.361 eV, respectively.^6^ Much of the research related to these new 2D carbon allotropes aims at studying their mechanical and electronic properties and their potential application as an anode material in lithium-ion batteries (LIB).

Y. Yu investigated the application of graphenylene as an anodic material in LIBs using DFT calculations.^7^ The adsorption of Li atoms in graphenylene was reported to be stronger than in pristine graphene, achieving a theoretical capacity of 1116 mA h g^–1^, which is greater than the capacity of graphene. Furthermore, due to the carbon rings (*C*_12_), diffusion paths were found with low-energy barriers (0.37–0.9 eV), allowing lithium atoms to be well dispersed on the graphenylene. Recently, Qiu et al.^8^ reported a theoretical capacity of 837 mA h g^–1^ and low diffusion barriers (0.36–0.83 eV) for a new 2D carbon allotrope composed of quadrangular, pentagonal, and hexagonal rings and large tetradecagonal pores (QPHT-graphene), which exhibits metallic behavior with a significant number of flat bands near the Fermi level region.^3,8^ Finally, there are other allotropes with promising high capacity and fast Li diffusion anode materials for LIBs, such as popgraphene^9^ and haeckelite h567,^10^ with theoretical capacities of 1487 mA h g^–1^ and 697 mA h g^–1^, respectively, as well as low Li diffusion barriers of (0.37–0.55 eV) and (0.21–0.35 eV).

The essential requirements for the application of a material as an anode of a LIB are good thermal stability to not induce structural deformations, high porosity values that are generally satisfied by high-order carbon rings (*C*_> 10_), and high values for electrical conductivity.^11^ The vast majority of these new 2D carbon allotropes have good thermal stability, because of strong C–C covalent bonds and high porosity values. However, they can also have large electronic band gap values, negatively influencing their application in LIBs. For example, pentagraphene, formed by *C*_5_ rings, has a theoretical capacity of 1489 mA h g^–1^, but has a band gap value of ∼3.25 eV.^12^ Furthermore, a theoretical capacity of 3916 mA h g^–1^ was reported for Twin-graphene, formed by *C*_6_ rings, which is the largest reported so far.^13^ Twin-graphene has semiconductor behavior with a band gap value of 0.75 eV.^14^

Tetra-penta-deca-hexagonal-graphene (TPDH-gr) was recently proposed by Bhattacharya and Jana.^15^ It is a 2D carbon allotrope composed of fully *sp*^2^ carbon atoms arranged in *C*_4_, *C*_5_, *C*_10_, and *C*_6_ rings, which has a cohesive energy 0.46 eV higher than that of graphene, but lower compared to other 2D carbon allotropes such as Twin-graphene and graphdiyne, the latter being an experimentally realized allotrope.^16^ This demonstrates the thermodynamic stability and the possibility of synthesis of TPDH-gr.

The experimental route proposed by D. Bhattacharya and D. Jana is based on the skeleton of a fulvalene derivative that can act as a precursor monomer. The dehydrogenation of these monomers can lead to the formation of nanoribbons, which can then undergo simultaneous cycloaddition and dehydrogenation to form the TPDH-graphene structure.^15^

Compared with other 2D carbon allotropes, TPDH-gr possesses good electronic and mechanical properties that enhance its potential application in LIBs. TPDH-gr has a metallic nature, without any energy gaps in its electronic band structure, facilitating electrical conduction at the negative electrode in LIBs. Additionally, TPDH-gr shows high thermal stability and anisotropic elastic properties due to its carbon ring arrangement topology.^15^ It exhibits a higher Young’s modulus than graphene along certain directions, which can translate into greater durability and resistance to mechanical degradation during charge–discharge cycles, a crucial aspect of the LIB lifetime.

It was recently reported that the *C*_4_ rings of TPDH-gr are more reactive and capable of adsorbing a larger amount of hydrogen than their other carbon rings.^17^ Furthermore, Oliveira et al. demonstrated that hydrogenation of TPDH-gr at the *C*_4_ rings also results in anisotropic thermoelectric properties.^18^ These recent works show TPDH-gr as a 2D carbon allotrope with interesting physical and chemical properties, motivating the investigation of its application as a LIB anode material.

In this work, the potential application of TPDH-gr as a LIB anode material was investigated using density functional theory (DFT)-based simulations. Through adsorption calculations, a high theoretical capacity of 1116 mA h g^–1^ for TPDH-gr was estimated, and lower diffusion barriers (0.08–0.2 eV) were found for Li atoms, comparable to the energy barriers of graphite, which is the most commercially used LIB anode.

## Computational Methods

The SIESTA software^19,20^ was utilized for all DFT simulations. For all calculations, a 2 × 1 supercell of TPDH-gr was considered. Periodic boundary conditions were applied to mimic infinitely large systems. A vacuum space of 20 Å was set to prevent spurious interactions between the layer and its periodic images. Within SIESTA, the Kohn–Sham orbitals were expanded using a double-ζ basis set consisting of numerical pseudoatomic orbitals of finite range, augmented with polarization orbitals. Optimized pseudopotentials and atomic bases from the SIMUNE database^21^ were employed with the Perdew–Burke–Ernzerhof (PBE) approximation for the exchange and correlation functional. Furthermore, van der Waals interaction corrections, equivalent to DF1,^22^ were incorporated into standard DFT calculations. The Brillouin Zone (BZ) sampling utilized a 5 × 8 × 1 irreducible Monkhorst–Pack (MP) k-point grid,^23^ for both TPDH-gr monolayer and bilayer calculations. The total energy convergence threshold for each electronic calculation was set at 1 × 10^–4^ eV. Geometry optimizations were performed using the conjugate gradient (CG) algorithm, ensuring that the magnitude of total forces acting on each ion was minimized to less than 0.01 Å/eV via ionic position displacements. Additionally, Bader charge analysis was carried out to quantify the charge transfer of lithium atoms.^24^

**Figure 1:**
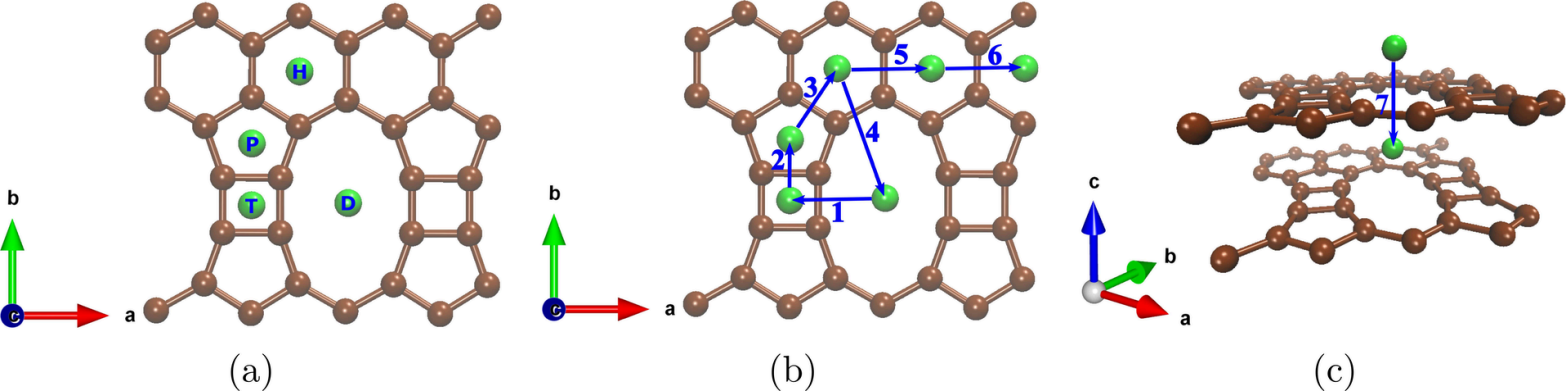
(a) Selected TPDH-gr regions for Li adsorption. (b) Investigated diffusion paths of lithium ions on the TPDH-gr monolayer. (c) Investigated diffusion path in a TPDH-gr bilayer. These paths were chosen based on minimum energy pathways. See text for discussions.

We calculated the adsorption energy (*E*_ads_) of a lithium atom on TPDH-gr layer using the following equation:
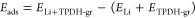
1where *E*_Li+TPDH-gr_ is the total energy of the final configuration of the Li atom adsorption process, while *E*_Li_ and *E*_TPDH-gr_ are the total energy of a gas system of Li and TPDH-gr. An alternative definition of the adsorption energy *E*_*ads*_^*bcc*^ was also considered, where the total energy per atom of lithium in bulk form (bcc) is considered. For both definitions of adsorption energy, negative values indicate that the Li atom is energy favorably adsorbed on TPDH-gr, while positive values indicate no adsorption. As shown in Figure 1, four adsorption regions (centers of the different rings) were considered: tetragonal (T), pentagonal (P), decagonal (D), and hexagonal (H) sites.

Adsorption calculations were carried out for the (N = 1, 2, ..., 6) Li atoms to find the storage capacity limit of TPDH-gr. For each value of N, 10 random different configurations were considered in which the distances between the lithium atoms were greater than 2.80 Å which is slightly larger than the molecular distance of Li_2_ (2.60 Å), to prevent Li clustering.^25^ These new *E*_*ads*_(*N*) are calculated using the following formula:
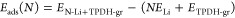
2where *E*_N–Li+TPDH-gr_ is the total energy of TPDH-gr with *N* adsorbed Li atoms. Through these calculations, it is possible to determine the maximum number of *N* Li atoms that can be absorbed, which is where the adsorption energy becomes positive.^25^ Therefore, once the value of *N* has been determined, it is easy to estimate the theoretical capacity of TPDH-gr using the following formula:
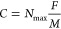
3where *N*_max_ is the maximum number of Li atoms adsorbed on TPDH-gr, *F* = 96485.332 s A/mol is the Faraday constant, and *M* is the molar mass of the TPDH-gr supercell.

Another important feature of anode material is the average open-circuit voltage (OCV), which is generally defined as the voltage between the terminals of an electrochemical cell when no current flows through the cell.^26,27^ The OCV can be calculated using the following equation:
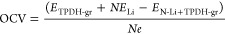
4where *e* is the electronic charge of the lithium ion. As seen in the previous equation, the OCV is related to the adsorption energy of the lithium atom. Thus, OCV negative values indicate that the adsorption is unfavorable, and the Li atoms will tend to form clusters. In contrast, positive values indicate the possibility of intercalation of lithium atoms, which further suggests good cycling performance.^28−30^

To better understand the charge transfer, we computed the charge density differences for Li adsorbed on TPDH-Graphene. These differences were determined using the following equation^26,27,31^
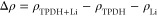
5where ρ_TPDH+Li_, ρ_TPDH_ and ρ_Li_ are the charge densities of Li adsorbed on TPDH-gr, pristine TPDH-gr and isolated Li atom, respectively.

To characterize Li diffusion on the surface of the TPDH-gr sheet, the minimum energy pathway (MEP) was calculated using nudged elastic band (NEB) calculations.^32,33^ The NEB method was discretized into five images in the configuration space, with fictitious springs connected between the images to prevent them from converging to the same local minima. Initially, images were linearly interpolated between the reactive and product states of the reaction and then optimized using a conjugate gradient method. A climbing image scheme (CI-NEB)^34^ was implemented to ensure an accurate determination of the transition state (TS). Seven diffusion paths for the Li atom were considered, as shown in Figure 1b,c.

For a better understanding of the Li diffusion process on TPDH-gr, we calculated the diffusion coefficients for the first 6 diffusion pathways. To achieve this, the Arrhenius equation was employed:^14^
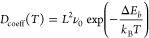
6where Δ*E*_*b*_ is the value of the diffusion barriers, *L* is the length of the diffusion path for the Li atom, *T* is the absolute temperature in kelvin units, ν_0_ is the vibration frequency, which usually has a value of 10 THz, and *k*_*B*_ is the Boltzmann constant (8.62 × 10^–5^ eV/K).^14^

## Results and Discussion

In Table 1, we present the values of *E*_*ads*_, *E*_*ads*_^*bcc*^, and the perpendicular distance *d*_*ads*_ from the Li adsorbed atom to the TPDH-gr layer. The adsorption calculations were carried out for a monolayer and a bilayer of TPDH-gr. These results show that the adsorption of Li on TPDH-gr is thermodynamically favorable, with negative values, and that the sites D and P are the preferential adsorption regions. On the other hand, the values of *E*_*ads*_^*bcc*^ are also negative; however, these values are smaller than of *E*_*ads*_. Additionally, the TPDH-gr bilayer presents adsorption energy slightly higher than the monolayer. However, it is also shown that the vdW correction (DF1) has a small effect on the Li adsorption energies and geometries.

**Table 1:** Adsorption Energy Values (*E*_ads_ and *E*_ads_^bcc^) and Final Distance (*d*_ads_) of the Li Atom at the Different Adsorption Regions (D, T, P, and H)^a^

	monolayer				bilayer			
	D	T	P	H	D	T	P	H
*E*_ads_	–2.59	–2.55	–2.59	–2.55	–2.65	–2.63	–2.67	–2.63
*E*_ads_^bcc^	–0.70	–0.66	–0.69	–0.66	–0.76	–0.74	–0.78	–0.74
*d*_ads_	1.46	1.90	1.82	1.77	1.46	1.86	1.82	1.76
*E*_ads_ (DF1)	–2.35	–2.30	–2.32	–2.27	–2.44	–2.41	–2.43	–2.39
*E*_ads_^bcc^ (DF1)	–0.68	–0.63	–0.65	–0.60	–0.77	–0.74	–0.76	–0.72
*d*_ads_ (DF1)	1.38	1.91	1.86	1.81	1.49	1.95	1.88	1.83

a For the adsorption process, TPDH-gr monolayers and bilayers were considered. van der Waals corrections (DF1) were also considered. The energies are given in eV, and the distances are in Å.

**Figure 2:**
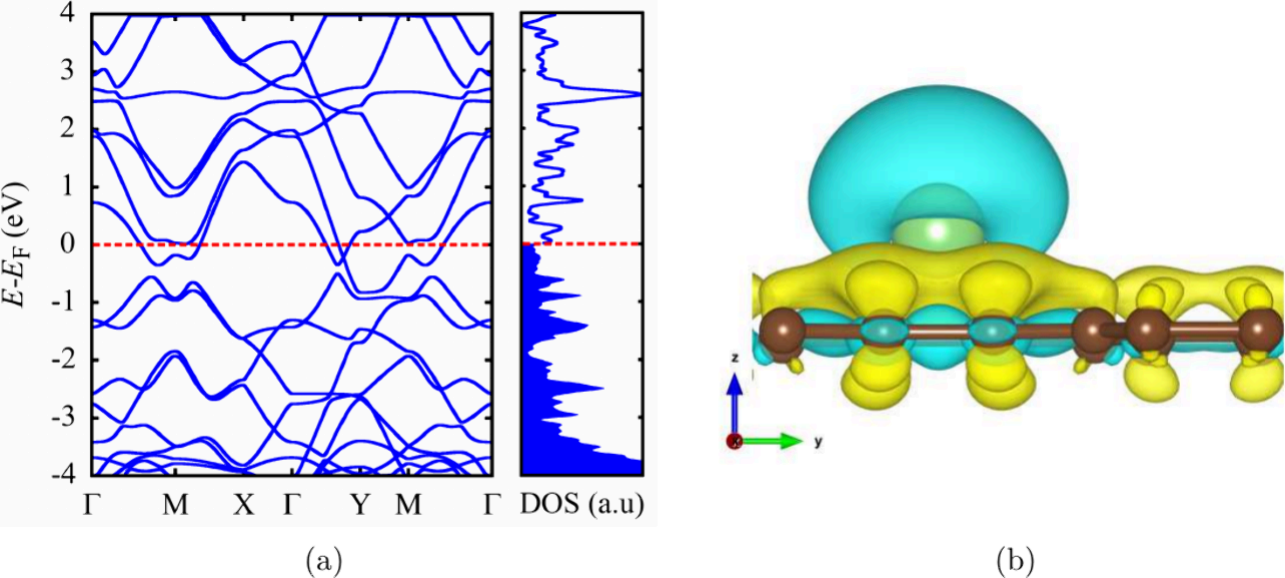
(a) TPDH-gr electronic band structure after the adsorption of Li on the D region. (b) Charge density difference for the adsorption of a single Li atom on TPDH-gr in the D region. The cyan and yellow regions represent the electron losses and gains, respectively. Here, the net charge density difference is defined as the Li-adsorbed TPDH-gr charge density minus the isolated Li atom and TPDH-gr charge density.

In Figure 2, we present the electronic band structure and the density of electronic states for the final configuration of TPDH-gr with a lithium atom adsorbed in the D region. As can be seen, the TPDH + Li system does not have a band gap because of the intrinsic metallic nature of TPDH-gr. To gain a deeper understanding of the adsorption properties between the Li atom and TPDH-gr, we performed calculations of the charge density differences (eq 5) for the D adsorption region, as shown in Figure 2. Our analysis revealed that electrons mainly accumulated around the carbon atoms, a result attributed to the higher electronegativity of carbon compared to lithium.

**Figure 3:**
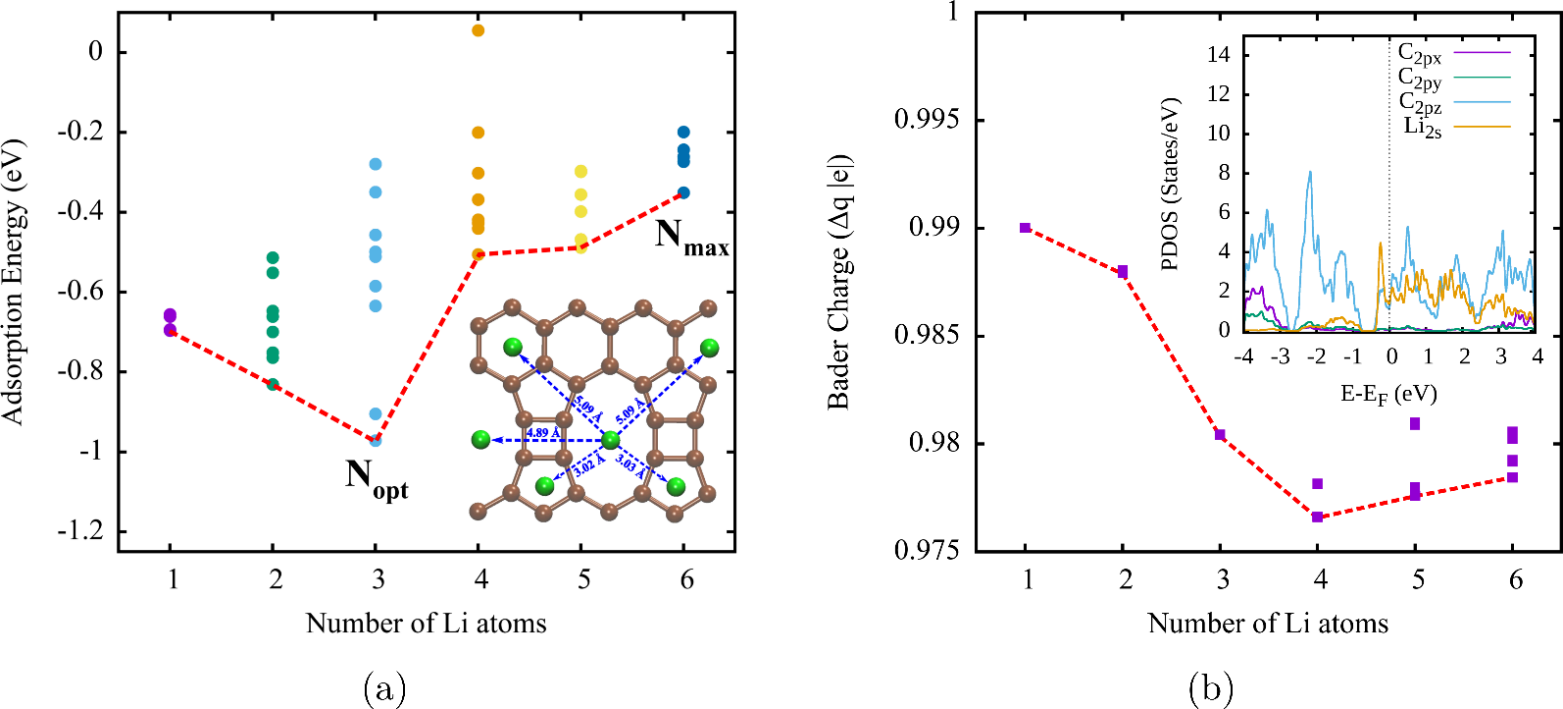
(a) Adsorption energies *E*_ads_^bcc^ (eV) as a function of the number *N* (*N* = 1, 2, ..., 6) of Li atoms. Inset: Configuration of TPDH-gr with (*N* = 6) Li atoms, showing that the distance between Li atoms is >2.80 Å to prevent clustering. (b) Bader charge as a function of the number of adsorbed lithium atoms on TPDH-gr. The inserted figure corresponds to the density of partial states for (*N* = 6) adsorbed lithium atoms.

The adsorption energy values *E*_*ads*_^*bcc*^ for Li atoms (N = 1, 2, ..., 6) are presented in Figure 3, where we connect the most stable configurations with a dotted line. It is clear that N = 3 is the optimal amount of Li atoms adsorbed on TPDH-gr, with a maximum value of *E*_*ads*_^*bcc*^ ∼ −1.0 eV. In Figure 3, it can also be seen that the maximum amount of Li that can be stored is N = 6, which only allows a maximum of 7 unique configurations with Li atoms separated by >2.8 Å (see inset in Figure 3). Considering both TPDH-gr sides, we have N = 12 as the maximum limit of lithium atoms adsorbed on the TPDH-gr. With these results, it is possible to calculate the theoretical capacity of TPDH-gr (for storage of Li atoms), following eq 3. The C value obtained was 1116 mA h/g. This capacity value is three times larger than the theoretical capacity of graphite (C = 372 mA h/g),^35^ which is the anode most used in commercial LIB and is comparable to the theoretical capacity of several 2D carbon allotropes: graphenylene (1116 mA h/g), popgraphene (1487 mA h/g) and Twin-graphene (3916 mA h/g),^36^ as can be seen in Table 2.

**Table 2:** Comparison of Various 2D Carbon Allotropes Showing Their Adsorption Energies (*E*_ads_), Specific Capacities (mAh/g), Diffusion Barriers (eV), Open-Circuit Voltages (V), and Their Electronic Nature (Metallic or Semiconductor)^a^

allotrope	*E*_ads_ (eV)	capacity (mAh/g)	diffusion barrier (eV)	OCV (V)	electronic nature
graphene		568^37^	0.23^38^		metallic
graphite		372^13^	0.22–0.40^39^	0.11^40^	metallic
graphenylene^7^	–0.56	1116	0.37–0.57		semiconductor
QPHT-graphene^8^		837	0.36–0.83	0.66	metallic
pop-graphene^9^		1487	0.37–0.55	0.45	metallic
twin-graphene^14^	–1.40	3916	0.22–0.53	0.32	semiconductor
graphdiyne	–2.63^41^	520^41^	0.18–0.84^42^		semiconductor
haeckelite^10^	–0.44	697	0.21–0.35	0.30	metallic
TPDH-gr	–0.70	1116	0.08–0.20	0.29	metallic ★

a Star symbol (★) represent the results of this work.

Figure 3 shows the charge variation of lithium atoms during the lithiation process, demonstrating a decrease in charge as a function of the number of lithium atoms absorbed on TPDH-gr. This decrease in charge can be explained by the smaller distance between Li atoms, which occurs as the concentration of Li on TPDH-gr increases. The inset shows the PDOS for (N = 6) lithium atoms adsorbed on TPDH-gr. As can be observed, there is a higher density of states corresponding to lithium atoms at the Fermi level, indicating that the system still preserves its intrinsic metallic nature.

**Figure 4:**
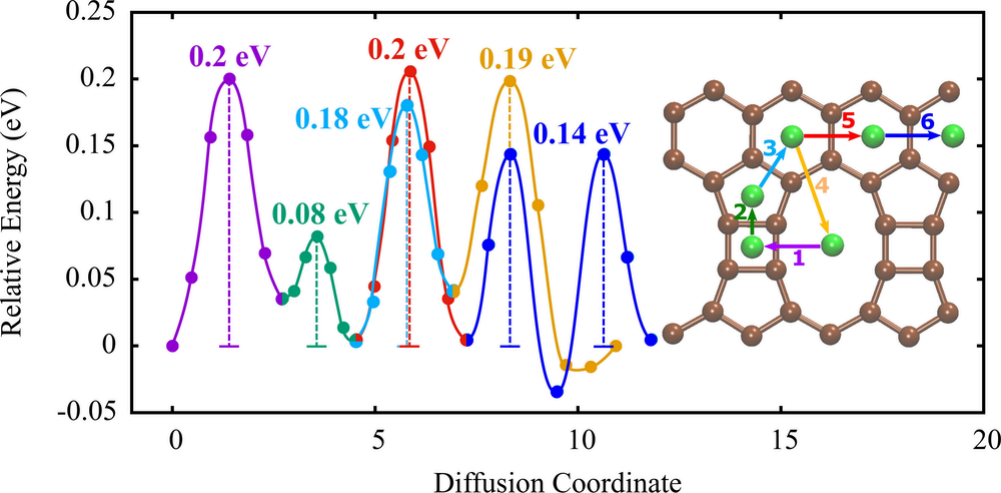
Diffusion energy barriers of the lithium atom on the surface of TPDH-gr. Inset: all intermediate Li images for each diffusion path are shown.

An important factor regarding the charge–discharge performance of a LIB is the diffusion of Li atoms on the surface of TPDH-gr. In Figure 4, the Li diffusion barriers for the six diffusion pathways considered in this study (see Figure 1) are shown, which were calculated using the NEB method. As can be observed from Figure 4, the energy barriers for Li diffusion have small values, with 0.2 eV being the maximum value for diffusion through pathways 1 and 5. Furthermore, the diffusion barriers for pathways 2, 3, 4, and 6 concerning pathway 1 have the following values: 0.08, 0.18, 0.19, and 0.14 eV, respectively. The diffusion barrier values for some 2D carbon allotropes are shown in Table 2: graphite (0.22–0.40 eV), haeckelite (0.21–0.35 eV), and Pop-graphene (0.37–0.55 eV). Therefore, because of the small diffusion barrier value of TPDH-gr, we can deduce that it could potentially exhibit equal or better performance in the charge and discharge process compared to graphene and graphite, which are currently the most widely used anodic materials for LIBs.

TPDH-gr is a material with anisotropic mechanical properties due to its specific distribution of *C*_4_, *C*_5_, *C*_6_, and *C*_10_ rings. Consequently, the anisotropy of TPDH-gr could extend to other physical and chemical properties, such as the diffusion of Li over TPDH-gr. As shown in Figure 4, diffusion paths 1, 5, and 6 represent horizontal diffusion, while paths 2, 3, and 4 represent vertical diffusion. The energy barrier values are not much different for the x and y directions.

**Figure 5:**
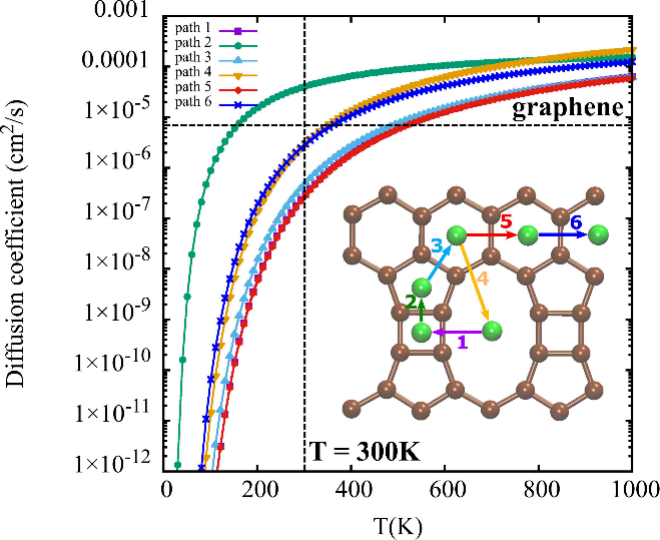
Diffusivity of Li atom over a TPDH-gr as a function of temperature (*T*). Inset: The four diffusion paths of Li on the TPDH-gr are shown.

To gain a deeper understanding of the Li diffusion process on TPDH-gr, we calculated the diffusion coefficients for the first six diffusion pathways using the Arrhenius equation (eq 6). In Figure 5, the diffusion coefficient is shown as a function of global temperature for the Li diffusion pathways in TPDH-gr. As observed, the *D*_coeff_ values at T = 300 K vary, with pathways 1, 3, and 5 showing slower Li diffusion. In contrast, the *D*_coeff_ of Li in pathways 2, 4, and 6 have larger values, indicating faster diffusion, even faster than diffusion in graphene (6 × 10^–6^ cm^2^/s).^14^

**Figure 6:**
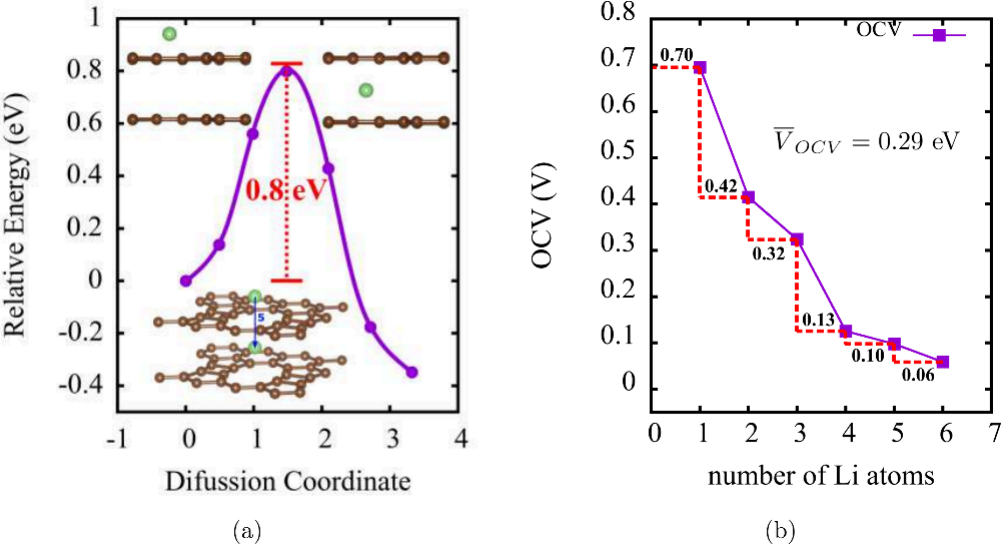
(a) Diffusion barrier of the lithium atom into a TPDH-gr bilayer. These interlayer diffusions were considered only in the D region. (b) Open circuit voltage (OCV) as a function of the number (*L*) of Li atoms adsorbed on TPDH-gr.

The Li diffusion barrier for a bilayer TPDH-gr is shown in Figure 6, where the diffusion path corresponds to the migration of the Li atom from the top (region D) to the middle of TPDH-gr, as can be seen in Figure 1. The diffusion barrier has a value of 0.8 eV, which indicates that Li diffusion occurs more easily on the surface of the TPDH-gr than along the diffusion path between layers. In Figure 6, the OCV is presented as a function of the number of Li atoms adsorbed in TPDH-gr. As observed in eq 4, the OCV correlates with the value of the adsorption energy of the Li atoms. Thus, small OCV values indicate moderate Li adsorption, allowing for easy Li desorption. The average OCV value shown in Figure 6 is 0.29 V, which is very close to the OCV values of other 2D carbon allotropes considered for application as anodes in LIBs: Pop-graphene (0.45 V) and Twin-graphene (0.32 V),^25^ as can be seen in Table 2.

## Conclusions

In conclusion, we have explored the potential of TPDH-gr as an anode material in LIBs using DFT-based calculations. Our results indicate that Li-atom adsorption on TPDH-gr is moderate (approximately −0.70 eV), facilitating easy desorption. Additionally, TPDH-gr exhibits an estimated high capacity of 1116 mAh/g, positioning it among the few 2D carbon allotropes with metallic properties and high capacity. Furthermore, the energy barriers (0.08–0.20 eV), diffusion coefficients, and Open Circuit Voltage (0.29 V) suggest rapid Li-atom diffusion on the surface, stable intercalation of Li atoms, and robust performance during the charge and discharge cycles of the LIB. These results, particularly the rapid diffusion of Li over TPDH-gr demonstrated in this work, indicate that TPDH-gr has a performance that is better or comparable to many other 2D carbon allotropes, such as pop-graphene, twin-graphene, and Haeckelite. These findings highlight TPDH-gr as a promising candidate for anode material in LIBs.
